# Learning view invariant recognition with partially occluded objects

**DOI:** 10.3389/fncom.2012.00048

**Published:** 2012-07-25

**Authors:** James M. Tromans, Irina Higgins, Simon M. Stringer

**Affiliations:** Experimental Psychology, Oxford Foundation for Theoretical Neuroscience and Artificial Intelligence, University of OxfordOxford, UK

**Keywords:** object recognition, continuous transformation, occlusion, inferior temporal cortex

## Abstract

This paper investigates how a neural network model of the ventral visual pathway, VisNet, can form separate view invariant representations of a number of objects seen rotating together. In particular, in the current work one of the rotating objects is always partially occluded by the other objects present during training. A key challenge for the model is to link together the separate partial views of the occluded object into a single view invariant representation of that object. We show how this can be achieved by Continuous Transformation (CT) learning, which relies on spatial similarity between successive views of each object. After training, the network had developed cells in the output layer which had learned to respond invariantly to particular objects over most or all views, with each cell responding to only one object. All objects, including the partially occluded object, were individually represented by a unique subset of output cells.

## 1. Introduction

It is important to understand how invariant representations of individual objects are built in the primate visual system even when multiple objects are present in natural scenes. Neurophysiological research has provided substantial evidence showing that over successive stages, the visual system develops neurons that respond with view, size, and position (translation) invariance to objects or faces (Desimone, 1991; Tanaka et al., 1991; Rolls, 1992, 2000; Perrett and Oram, 1993; Rolls and Deco, 2002). For example, it has been shown that the inferior temporal visual cortex has neurons that respond to faces and objects with translation (Kobatake and Tanaka, 1994; Tovee et al., 1994; Ito et al., 1998; Op De Beeck and Vogels, 2000), and view (Hasselmo et al., 1989; Booth and Rolls, 1998) invariance.

The “biased competition hypothesis” of attention suggested that feedback connections are necessary to build separate representations of individual objects in a complex scene by providing the mechanism for attentional selection (Rolls and Deco, 2002). However, it has been shown that this separation can be achieved without the need for an attentional mechanism using purely feedforward connectivity in a hierarchical neural network model of the ventral visual pathway, VisNet (Stringer et al., 2007). The statistical properties of the input stimuli play a crucial role, whereby the features within individual objects occur more frequently together than the features between different objects. As such, although the role of feedback connections is an important area for future research, they will not be implemented in the present study.

Stringer and Rolls (2000) showed that a hierarchical neural network model of the ventral visual pathway, VisNet, could recognize objects presented against natural cluttered scenes, providing the model had been previously trained with each object presented individually transforming against a blank background. However, the network failed to learn to recognize individual objects if the objects were presented against a natural cluttered background during training.

Recent studies by Stringer and Rolls (2008) and Stringer et al. (2007) have shown how VisNet may cope with complex scenes during training, and learn invariant representations of individual objects even when no single object is seen in isolation. These modeling studies used the statistics of the natural environment where features within an object occur together more frequently than features between different objects. Specifically, VisNet could learn invariant representations of individual objects if different combinations of transforming objects were seen at different times.

However, a further challenge is to explain how invariant representations can be learned when the objects are partially occluded by one another during learning. Stringer et al. (2007) proposed that Continuous Transformation (CT) learning (Stringer et al., 2006) combined with the statistical independence of objects presented in different combinations might allow the network to solve this problem. Specifically, consider presenting a number of objects to the network in different subset combinations, but where one of the objects is always partially occluded by whichever objects it is currently shown with. The hypothesis is that the network will simultaneously form separate representations of all of the different objects, where an invariant representation of the partially occluded object is formed by linking together the different partial views through CT learning. However, Stringer et al. (2007) provided no simulation evidence that this could work. In this paper we demonstrate for the first time this process operating with simulated three dimensional rotating objects. It is important to investigate this issue because objects in the natural environment will often overlap. This task is more difficult than simply forming separate representations of different objects because, in order for the network to build a complete invariant representation of the partially occluded object, the network has to link together the different partial views of the object as well as separate these partial views from the other objects present.

In the simulations described below, we show how VisNet can form separate view invariant representations of individual objects seen rotating together, where one of the rotating objects is always partially occluded by the other objects present during training. The network develops cells in the output layer which have learned to respond invariantly to particular objects over most or all views, with each cell responding to only one object. All objects, including the partially occluded object, are individually represented in this way by a unique subset of output cells. This learning process relies on the statistical independence of the objects that are shown in different combinations, as well as an invariance learning mechanism known as CT learning that is described next.

## 2. Materials and methods

### 2.1. Continuous transformation learning

A leading computational theory of how the ventral visual pathway in the brain may develop neurons that respond to objects with transform (e.g., view or location) invariance is CT learning. CT learning uses an associative (Hebbian) synaptic modification rule (Stringer et al., 2006) that can exploit the image similarity across successive transforms (e.g., views) of a continuously transforming object in order to develop output neurons which respond to the object over all transforms. Because CT learning is based on the standard Hebbian learning rule, it is biologically plausible.

**Figure 1:**
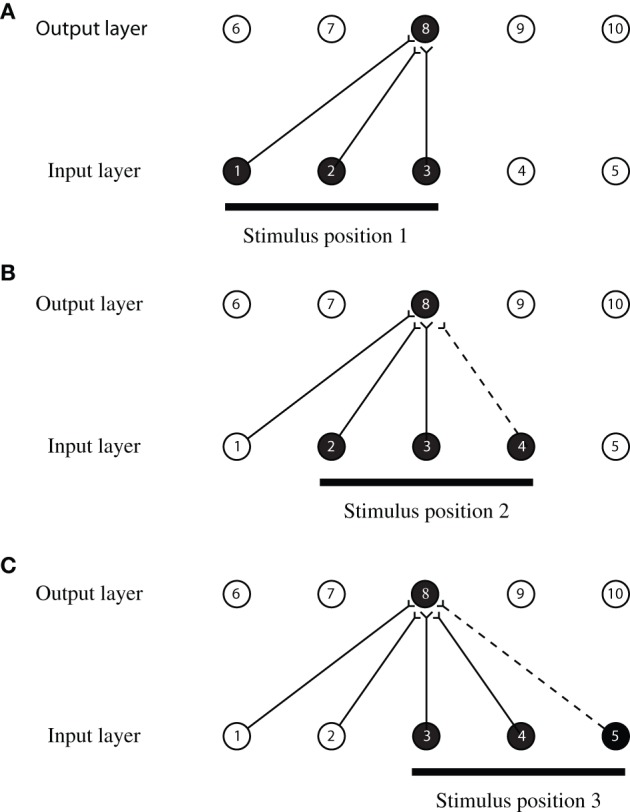
**An illustration of how CT learning functions in a feed-forward one-layer network.** Activation of overlapping neurons during the transformation of the object from position to position leads to the activation of the same neuron in the output layer. Connections are strengthened according to a Hebbian learning rule after each presentation of the stimulus.

An idealized version of the CT learning process outlining the theoretical principle is illustrated in Figure 1 and operates as follows. The network shown has an input layer where stimuli are presented, and an output layer where transform invariant representations develop through learning. The output layer operates as a competitive network, where individual cells send inhibitory projections to the other cells in this layer (not shown in Figure 1), and thereby compete with each other. Initially, the weights of the feedforward synaptic connections are set to random values. Then, during learning, a stimulus is initially presented in position 1 (shown in Figure 1A) and is represented by three active neurons in the input layer (neurons 1, 2, and 3). Activity propagates through the random feedforward connections to the output layer, where one of the neurons, say neuron 8, wins the competition. The simultaneous activation of neurons in the input and output layers causes the synaptic connections between them to become strengthened according to a Hebbian learning rule

(1) $$\delta w_{ij}=\alpha y_{i}x_{j}$$

where δ*w*_*ij*_ is the increment in the synaptic weight *w*_*ij*_, *y*_*i*_ is the firing rate of the post-synaptic neuron *i*, *x*_*j*_ is the firing rate of the pre-synaptic neuron *j*, and α is the learning rate. To restrict and limit the growth of each neuron's synaptic weight vector, *w*_*i*_ for the *i*th neuron, its length is normalized at the end of each timestep during training as is usual in competitive learning (Hertz et al., 1991). This is necessary to ensure that one or a few neurons do not always win the competition. If there was no normalization of synaptic weights during a simple Hebbian learning procedure, just a few neurons may eventually learn to respond strongly to nearly all of the input patterns. Neurophysiological evidence for synaptic weight normalization is provided by Royer and Pare (2003).

As the stimulus moves from position 1 to position 2 (shown in Figure 1B), it causes activation in the input layer to also move along one neuron at a time. Therefore, when the stimulus is in position 2, it causes neurons 2, 3, and 4 to become active. The overlap in the input space allows two neurons in the input layer to remain active (neurons 2 and 3) during both transformations. The activation of the same neurons in the input layer causes the same neuron in the output layer (neuron 8) to become active again because the connections have already been strengthened when the stimulus was in position 1. The simultaneous activation of the output neuron, with input neurons 2, 3, and the additional input neuron 4 causes their synaptic connections to become strengthened according to the Hebbian leaning rule. Therefore, the activation of neuron 8 will now become associated with the activation of neurons 2, 3, and 4. As the stimulus continues to move from one position to the next, the process repeats itself and the same neuron in the output layer remains activated. This output neuron becomes a position invariant neuron. A more comprehensive description of CT learning and simulation results in the context of invariant object recognition is provided by Stringer et al. (2006) and Perry et al. (2006).

### 2.2. Learned object selectivity

CT learning develops transform invariant representations that are object-specific. That is, as long as each object is not always presented together with another particular object transforming in lock-step during training, individual neurons typically learn to respond to one object only (Stringer et al., 2006). Consider an object rotating at a particular retinal location during training. Successive views of the object are represented by the outputs of the oriented input filters representing V1 simple cells as described in Equation 2. CT learning utilizes Hebbian competitive learning. At each presentation of a view of an object during training, activity is propagated up through successive neuronal layers in the network. Within each layer, a small subset of neurons wins the competition.

The feedforward synaptic connections from the input filters to the first layer of neurons are modified according to a Hebbian learning rule (Equation 1). This learning rule strengthens only the synaptic connections from those V1 filters that are activated by the particular visual form of the object view currently presented. The weight vector of each of the first layer neurons gradually shifts during learning to point in the same direction as the V1 input pattern(s) that it is learning to respond to. Since each first layer neuron computes its activation (Equation 3) according to the dot product of its weight vector and the current input pattern from the V1 input layer, after training each neuron will respond in proportion to the similarity between the current input pattern and the input pattern(s) the neuron learned to respond to during training. That is, each neuron will respond maximally to the input pattern that it has learned to respond to during training, and generalize to other input patterns depending on their similarity (Hertz et al., 1991).

The competitive Hebbian learning rule operates in a similar manner for the feedforward connections between all of the later layers of the network. This ensures that a subset of neurons in layer 1 and all successive layers learn to respond to the pattern of visual features present in the current view of the trained object. This means that the subset of output neurons in the higher layers of VisNet learn to respond to the visual form of the current view of the trained object and not its retinal location.

If output neurons simply learned to respond to retinal location, then the feedforward connections would need to be strengthened from *all* of the V1 filter inputs in a particular location regardless of the visual form of the objects. But this cannot occur because the Hebbian learning rule ensures that only the synaptic connections coming from those V1 input filters actually activated by the particular visual form of the object can be strengthened.

As described above in the Materials and Methods section on CT learning, the Hebbian learning rule is able to learn to associate different views of the object onto the same active output neurons as long as the different object images presented during training cover a space of smoothly changing views. Again, only those V1 input filters that were activated by the different object views can become associated with the active subset of neurons in the higher layers. So, even after many stimulus views have been presented, the neurons in the later layers of the network cannot learn to respond to all of the V1 filters in a particular retinal location. Thus, after training, the output neurons become object-specific. The output neurons will respond maximally to different views of the particular object that has been learned.

If another different untrained object is presented in the same retinal location as the first trained object, then there will be a rather different pattern of V1 input filters activated. However, as discussed above, the output of each of the neurons in the network reflects the similarity between the input pattern in the previous layer that it learned to respond to during training and the currently tested input pattern. This means that the neurons in the higher layers that have been previously trained to respond to the first object will respond to the second untrained object in proportion to the degree of visual similarity between the two objects. Therefore, due to the properties of Hebbian competitive learning, the neurons through the higher layers of the network must learn to respond the visual forms of objects rather than locations.

However, there is a potential conflict between the need to develop representations that are object-specific and the need to develop transform invariant representations within each object. In principle, if two different objects have similar transforms, then the CT learning mechanism may encourage output neurons to learn to respond invariantly across both objects. This is a fundamental issue with CT learning, which we are continuing to investigate. In simulation studies, we have found that increasing the size of the VisNet architecture improves the ability of the model to learn separate representations of similar faces for example. It is also possible that combining CT learning with a trace learning rule (Foldiak, 1991) could improve the ability of the network to form separate invariant representations of different objects. Although, this has not been implemented in the simulations reported here, which use only a standard Hebbian learning rule.

### 2.3. Multiple objects

How the brain can build invariant representations of individual objects even when multiple objects are present in a scene is a very important question in natural vision. How the visual system learns about individual objects rather than the combination of objects that make up the scene has only recently been investigated successfully in a biologically realistic model (Stringer et al., 2007; Stringer and Rolls, 2008). The features that make up a given object occur together more frequently when presented during training compared to the features that make up different objects. Depending on how often a given object is presented during training with another object, the frequency of how often features between these two different objects occur together will vary. However, the features that make up any individual object are always presented with one another and are therefore completely correlated.

It has been shown that a competitive network will operate usefully in this situation. The network will learn primarily to form representations that reflect the high probability of co-occurrence of features from one object and do not reflect the features of other objects presented simultaneously during training if the object being trained is seen much more frequently than it is presented with any other object.

In order for a competitive network to build representations of individual objects, there must be a statistical decoupling between the features that comprise each of the objects presented during training. Providing that there are a sufficient number of objects present during training, each object will be presented with many other objects and therefore the features within the object will appear together significantly more often than they are coupled with features of any other object. It has been previously demonstrated that this allows a competitive network to form transform invariant representations of individual objects, rather than the combinations of objects seen during training, by a mechanism such as CT learning (Stringer and Rolls, 2008).

### 2.4. Learning to recognise partially occluded transforming objects

The major new problem addressed in this paper is how VisNet can form separate view invariant representations of individual objects seen rotating together, where one of the rotating objects is always partially occluded by the other objects present during training. To create view invariant representations of the occluded object, the network will have to separate it from the occluding objects and link together different partial views to create a representation of the whole object. The potential solution described by Stringer and Rolls (2008) for separating out individual objects in a scene with multiple objects present will be used to separate the occluded and the occluding objects. CT learning will be used to link together the different transforms of each individual object, including associating together the occluded and unoccluded views. By training VisNet with multiple objects that partially occlude one another, we show that our model of the ventral visual stream is able to learn reliably in increasingly realistic visual environments.

### 2.5. Objects

**Figure 2:**
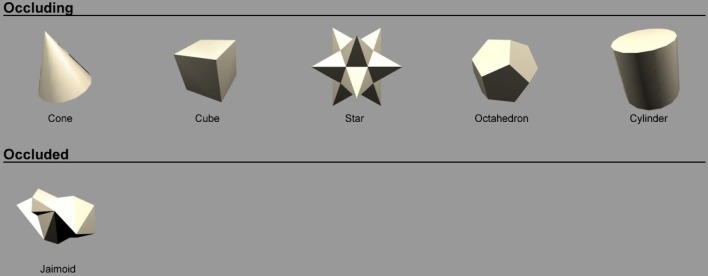
**The six objects used to train the network.** The objects are 3D objects each shown from 360 different views. The effect of the ambient lighting and single diffuse light source is illustrated. This allows different surfaces to be shown with different intensities. Objects are split into two groups; occluding objects are presented in the top row and the occluded object is presented in the bottom row.

Figure 2 shows the objects used to train the network. There were *N* = 6 continuously rotating 3D objects on a gray background. Previous research (Stringer and Rolls, 2008) has shown that *N* = 6 objects is sufficient to allow VisNet to develop representations of individual objects when the network was trained on object pairs. The objects were designed and created using the 3D modeling tool Swift 3D 5.4. Ambient lighting with a diffuse light source was added to allow different surfaces to be shown with different intensities. Each object rotated in depth around the vertical axis in 1° steps over 360°. This step size was chosen because past research (Stringer et al., 2006) has revealed that it was sufficiently small for CT learning to operate. The 360 views of each object were then exported as 2D JPG images and encapsulated as Adobe Shock Wave Files. The objects were then aligned and organized using Adobe Flash CS4.

**Figure 3:**
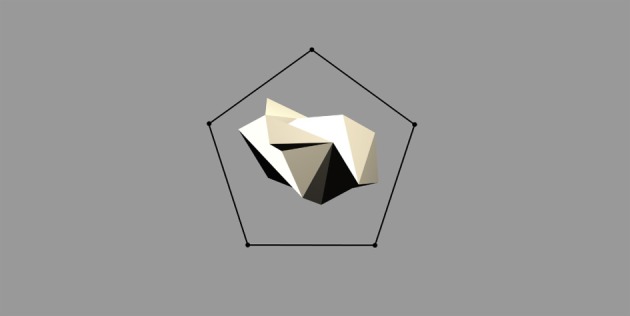
**The pentagon formation specifying the location of the occluding and occluded objects.** The occluded object, the Jaimoid, is always presented in the centre of the pentagon. Each of the occluding objects is placed at one of the five points of the pentagon, partially overlapping the Jaimoid at the center. This ensures that the occluding objects are equidistant from the center of the occluded object, helping to maintain a comparable level of partial occlusion for the Jaimoid.

**Figure 4:**
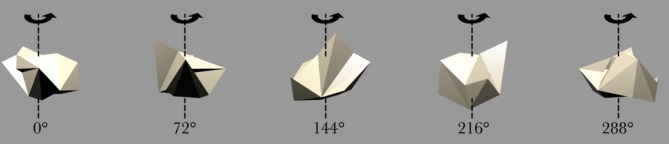
**Five example frames selected from the 360 frame testing image sequence of the Jaimoid rotating in depth around the vertical axis through 360° in 1° steps.** The selected frames shown are for 0°, 72°, 144°, 218°, and 288°. All five of the occluding objects were also presented to the network in the same manner.

The stimulus set was comprized of five occluding objects and one occluded object. During each training sequence, the occluded object was shown rotating with one of the occluding objects. In all cases, each object would rotate about its own vertical axis and, therefore, all axes were in parallel with one another. The spatial arrangement of the objects is shown in Figure 3. The occluding objects were presented in a pentagon formation. The occluded object, the Jaimoid (irregular multifaceted three dimensional object, Figure 4), was always presented in the center of the pentagon.

Each of the occluding objects was placed at one of the five points of the pentagon, partially overlapping the Jaimoid at the center. The occluding objects were equidistant from the center of the occluded object, therefore occluding it to the same extent. The occluded object was always behind the occluding objects and in the middle of the pentagon formation. This spatial formation was chosen because it was necessary to ensure that different parts of the occluded object were covered by the five occluding objects.

In our simulations the objects were rotating at the same speeds. However, the correlations that would arise between corresponding view points of the objects are broken due to the fact that objects are paired with different objects on different occasions. This allows the network to form separate representations of different objects in the output layer.

### 2.6. The VisNet model

The model architecture (VisNet) implemented by Wallis et al. (1993) and Wallis and Rolls (1997) that is used to investigate the properties of CT learning in this paper is based on the following: (1) A series of hierarchical competitive networks with local graded inhibition. (2) Convergent connections to each neuron from a topologically corresponding region of the preceding layer, leading to an increase in the receptive field size of neurons through the visual processing areas. (3) Synaptic plasticity based on a Hebb-like learning rule. Model simulations which incorporated these hypotheses with a modified associative learning rule to incorporate a short term memory trace of previous neuronal activity (Foldiak, 1991) were shown to be capable of producing object-selective but translation and view invariant representations (Wallis and Rolls, 1997; Rolls and Milward, 2000; Rolls and Stringer, 2001).

CT learning and trace learning are two biologically plausible learning mechanisms that have been used to model invariance learning in the visual system. Each may explain how neurons at the end of the ventral visual pathway learn to respond to visual stimuli with transform (e.g., position or view) invariance. With CT learning (Stringer et al., 2006), a standard Hebb learning rule is able to encourage output neurons to learn to respond invariantly across different transforms of an object. CT learning utilizes the spatial overlap or similarity between different transforms of an object in order to produce invariant responses. In contrast, the trace learning rule (Foldiak, 1991) incorporates a memory trace of recent neuronal activity, which is able to exploit the temporal continuity of the different transforms of an object in order to produce invariant responses. The trace learning rule assumes that in the natural visual world different transforms of an object tend to occur close together in time. In this paper, we will explore only the performance of the CT learning mechanism, which relies on the simpler Hebb learning rule.

The CT learning principle in the model architecture (VisNet) uses only spatial continuity in the input objects to drive the Hebbian associative learning with no temporal trace. In principle, the CT learning mechanism we describe could operate in various forms of feedforward neural network, with different forms of associative learning rule or different ways of implementing competition between neurons within each layer.

**Figure 5:**
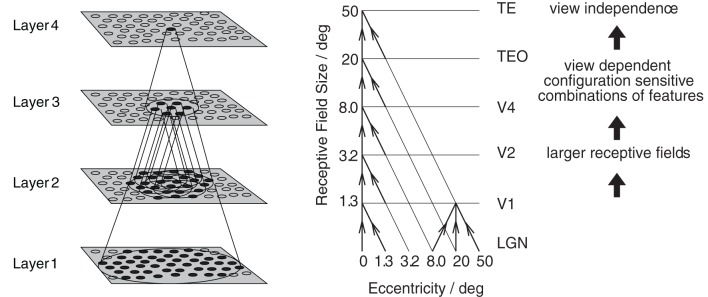
**Left:** Stylised image of the 4 layer network developed by Wallis et al. (1993) and Wallis and Rolls (1997). Convergence through the network is designed to provide fourth layer neurons with information from across the entire input retina. **Right:** Convergence in the visual system V1, visual cortex area V1, TEO, posterior inferior temporal cortex; TE, inferior temporal cortex (IT).

The model consists of a hierarchical series of four layers of competitive networks that are intended to model the hierarchy of processing areas in the ventral visual stream, which include V2, V4, the posterior inferior temporal cortex, and the anterior inferior temporal cortex, as shown in Figure 5. The forward connections to individual cells are derived from a topologically corresponding region of the preceding layer, using a Gaussian distribution of connection probabilities. These distributions are defined by a radius which will contain approximately 67% of the connections from the preceding layer. The values used are given in Table 1.

**Table 1:** **Network dimensions showing the number of connections per neuron and the radius in the preceding layer from which 67% are received**.

	**Dimensions**	**Number of connections**	**Radius**
Layer 4	32 × 32	100	12
Layer 3	32 × 32	100	9
Layer 2	32 × 32	100	6
Layer 1	32 × 32	272	6
Retina	128 × 128 × 32	−	−

**Figure 6:**
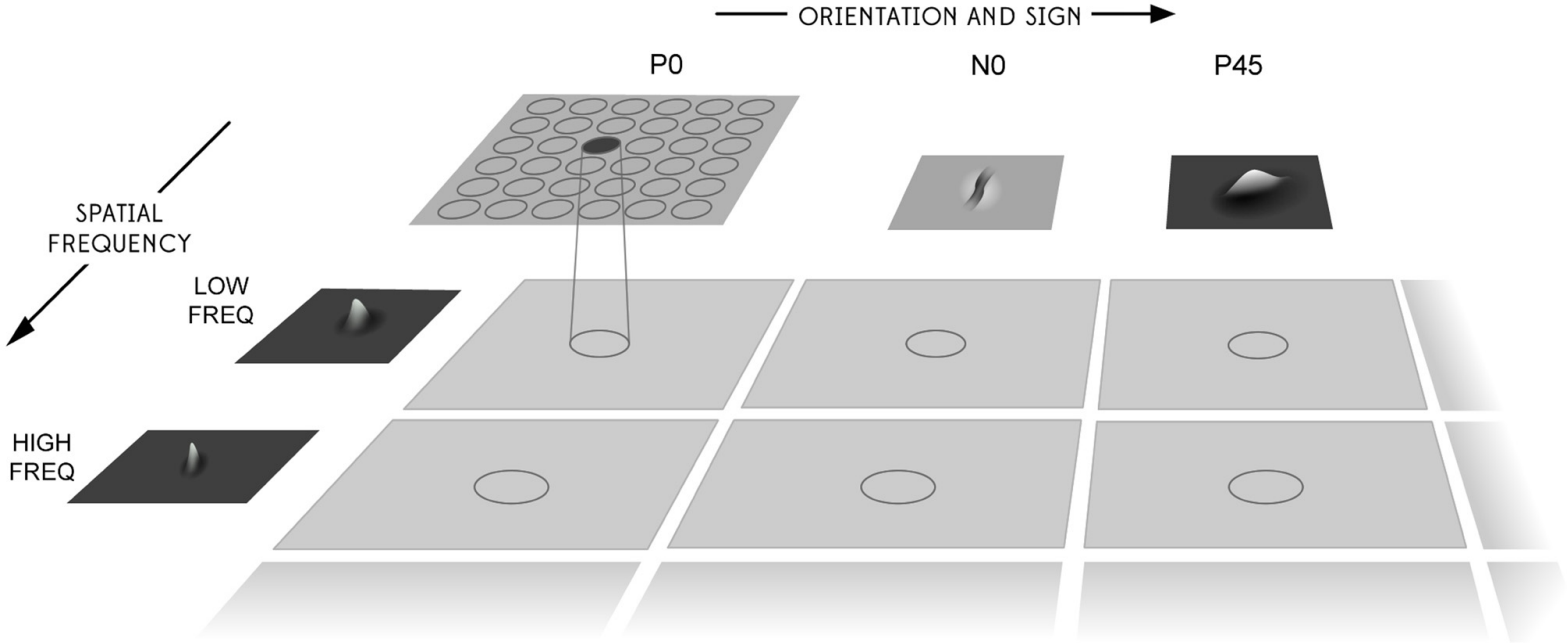
**The filter sampling paradigm.** Images are first filtered by a difference of Gaussian filter of the appropriate orientation, sign, and frequency. Each square represents the retinal image after filtering and the circles represent the consistent retinotopic coordinates used to provide input to a layer one cell. The orientation tuning, left to right, increases from 0° in steps of 45°, with segregated pairs of positive (P) and negative (N) filter responses. The filters double in spatial frequency toward the reader. For further details, see Rolls and Deco (2002).

Before the objects are presented to the network's input layer they are pre-processed by a set of input filters which accord with the general tuning profiles of simple cells in V1. The filters provide a unique pattern of filter outputs for each transform of each visual object, which is passed through to the first layer of VisNet. The input filters used are computed by weighting the difference of two Gaussians by a third orthogonal Gaussian according to the following:

(2) $$\Gamma_{xy}(\rho ,\theta ,f)=\rho \left[e^{-{\left(\frac{x\cos\theta +y\sin\theta }{\sqrt{2}/f}\right)}^{2}}-\frac{1}{1.6}e^{-{\left(\frac{x\cos\theta +y\sin\theta }{1.6\sqrt{2}/f}\right)}^{2}}\right]e^{-{\left(\frac{x\sin\theta -y\cos\theta }{3\sqrt{2}/f}\right)}^{2}}$$

where *f* is the filter spatial frequency, θ is the filter orientation, and ρ is the sign of the filter, i.e., ± 1. Individual filters are tuned to spatial frequency (0.0625–0.5 cycles/pixel); orientation (0°–135° in steps of 45°); and sign (±1). Example input filters are shown in Figure 6. In previous studies, we have found that four filter orientations θ is the minimal number needed to distinguish effectively between different visual objects presented to the retina. The number of layer 1 connections to each spatial frequency filter group is given in Table 2. Our model incorporates four octaves of filter frequencies. There are more connections from high frequency filters than low frequency filters. This enables the high frequency filters to cover a similar region of the input as the low frequency filters. Past neurophysiologcal research has shown that models based on difference-of-Gaussians functions are superior to those based on the Gabor function or the second differential of a Gaussian. Although the DOG-based models have more free parameters, they can account better for the variety of shapes of spatial contrast sensitivity functions observed in cortical cells and, unlike other models, they provide a detailed description of the organization of subregions of the receptive field that is consistent with the physiological constraints imposed by earlier stages in the visual pathway. (Hawken and Parker, 1987).

**Table 2:** **Layer 1 connectivity**.

Frequency	0.5	0.25	0.125	0.0625
Number of connections	201	50	13	8

The numbers of connections from each spatial frequency set of filters are shown. The spatial frequency is in cycles per pixel.

The activation *h*_*i*_ of each neuron *i* in the network is set equal to a linear sum of the inputs *y*_*j*_ from afferent neurons *j* weighted by the synaptic weights *w*_*ij*_. That is,

(3) $$h_{i}=\sum_{j}w_{ij}y_{j}$$

where *y*_*j*_ is the firing rate of neuron *j*, and *w*_*ij*_ is the strength of the synapse from neuron *j* to neuron *i*.

Within each layer, competition is graded rather than winner-take-all, and is implemented in two stages. First, to implement lateral inhibition, the activation *h* of neurons within a layer are convolved with a spatial filter, *I*, where δ controls the contrast and σ controls the width, and *a* and *b* index the distance away from the center of the filter

(4) $$I_{a,b}=\{\begin{array}{ll} -\delta e^{-\frac{a^{2+}b^{2}}{\sigma^{2}}} & \text{if}\;a\;\neq \;0\text{ or }b\;\neq \;0,\\ 1-\sum_{\begin{array}{c} a\;\neq \;0\\ b\;\neq \;0 \end{array}}I_{a,b} & \text{if}\;a\;=\;0\text{ and }b\;=\;0. \end{array}$$

The lateral inhibition parameters are given in Table 3.

**Table 3:** **Lateral inhibition parameters**.

Layer	1	2	3	4
Radius, σ	1.38	2.7	4.0	6.0
Contrast, δ	1.5	1.5	1.6	1.4

Next, contrast enhancement is applied by means of a sigmoid activation function

(5) $$y=f^{sigmoid}(r)=\frac{1}{1+e^{-2\beta (r-\alpha )}}$$

where *r* is the activation (or firing rate) after lateral inhibition, *y* is the firing rate after contrast enhancement, and α and β are the sigmoid threshold and slope respectively. The parameters α and β are constant within each layer, although α is adjusted to control the sparseness *a* of the firing rates. The sparseness *a* of the firing within a layer can be defined, by extending the binary notion of the proportion of neurons that are firing, as

(6) $$a=\frac{{\left(\sum_{i=1}^{N}y_{i}/N\right)}^{2}}{\sum_{i=1}^{N}y_{i}^{2}/N}$$

where *y*_*i*_ is the firing rate of the *i*th neuron in the set of *N* neurons (Rolls and Treves, 1990, 1998). For the simplified case of neurons with binarized firing rates = 0/1, the sparseness is the proportion ∈ [0, 1] of neurons that are active. To set the sparseness to, say, 5% in VisNet simulations, the threshold α is set to the value of the 95th percentile point of the activations within the layer.

The parameters for the sigmoid activation function are shown in Table 4. These fall squarely within the standard VisNet sigmoid parameter values which have been previously optimised to provide reliable and robust performance (Stringer et al., 2006, 2007; Stringer and Rolls, 2008).

**Table 4:** **Sigmoid parameters**.

Layer	1	2	3	4
Percentile	95	95	88	91
Slope β	190	40	75	26

### 2.7. Training procedure

The lateral inhibition and contrast enhancement stages of the VisNet model aim to simulate the function of inhibitory interneurons. In the brain, inhibitory interneurons effect direct competition between nearby excitatory cells within each layer of the ventral visual pathway. The way in which contrast enhancement is currently implemented in VisNet allows us to control the sparseness of firing rates within each layer. This is a useful aspect of the model, which allows us to explore the effects of sparseness on network performance. Although, it should be noted that the current contrast enhancement mechanism is not as realistic as implementing local inhibitory neurons explicitly because it is a global operation across each entire layer.

**Figure 7:**
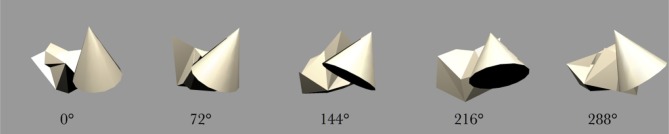
**Five example frames selected from the 360 frame training image sequence of the Jaimoid and the Cone rotating through 360° in 1° steps.** The selected frames shown are for 0°, 72°, 144°, 218°, and 288°. The Jaimoid is also occluded by the four other occluding objects in separate image sequences. Therefore, in total, there are five image sequences used during training, each containing 360 frames.

**Figure 8:**
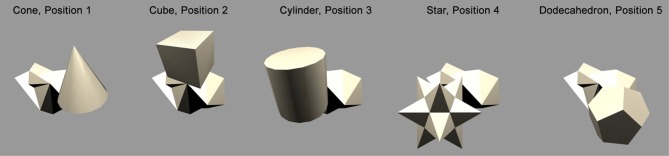
**Five example frames of the Jaimoid occluded by all five occluding objects in their five corresponding positions.** The occluding objects are arranged around the pentagon formation so that they are equidistant from the center of the Jaimoid. The rotating objects used during training are presented in the same locations: cone, position 1; cube, position 2; cylinder, position 3; star, position 4; dodecahedron, position 5; Jaimoid, position 6.

**Figure 9:**
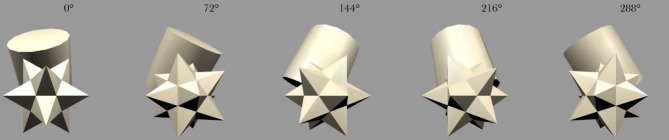
**Five example frames of two occluding stimuli (cylinder and star) rotating together, demostrating the typical overlap between the occluding objects**.

The occluded object, the Jaimoid, paired with each of the five surrounding occluding objects, is presented to VisNet with both objects rotating over 360° (Figure 7). Each full revolution over 360° of the pair is followed by the occluded object paired with a different occluding object in a different location around the pentagon formation. This process is repeated until the occluded object is paired with all five occluding objects. The rotating objects are presented as follows: cone, position 1; cube, position 2; cylinder, position 3; star, position 4; dodecahedron, position 5; Jaimoid, centrally, at position 6 (Figures 8 and 9).

In addition, all possible pairings of the five occluding objects are then presented in a similar fashion rotating over 360°. This helped VisNet to learn separate representations of the objects by using the statistics of the natural environment where the features within an object occur together more frequently than features between different objects (Stringer and Rolls, 2008). It should be noted that adjacent pairs of occluding objects would also sometimes overlap during training, leading to one occluding object being partially occluded by another occluding object.

At each image presentation, the activation of individual neurons within a layer is calculated, then their firing rates are calculated, and the feedforward synaptic weights between layers *w*_*ij*_ are updated according to Equation 1. This process is repeated for each layer in turn for all 4 layers of the VisNet model. One training epoch consists of the occluded object paired with all five occluding objects across all 360 transforms followed by all possible pairings of the occluding objects rotating over all 360°.

In this manner, the network is trained one layer at a time starting with layer 1 and finishing with layer 4. Fifty training epochs were used for layers 1–4. The learning rate for layers 1–4 were 0.109, 0.1, 0.1, and 0.1, respectively.

Due to high computational expense, the Jamoid was the only object that was partially occluded by its neighbours in the simulations described below. However, the underlying theory described above predicts that similar effects would be found if the simulations were repeated with more objects partially occluded by each other during training.

### 2.8. Testing procedure

During testing, the synaptic weights within the model are fixed and cannot be altered. Firstly, in order to test whether VisNet had built an invariant representation of the central partially occluded object, the occluded object was presented individually, rotating around the vertical axis over 360° (Figure 4). The surrounding five occluding objects were also presented in isolation in a similar fashion to verify that VisNet had build invariant representations of these objects too. The neuronal outputs of the network were then recorded during the testing presentations of each view of each object.

Secondly, VisNet was also tested with six novel objects rotating over 360° in 1° steps. This test demonstrates whether Visnet has learned to respond to the specific objects presented during training or whether VisNet has learned to respond selectively to only the location where these objects were presented.

**Figure 10:**
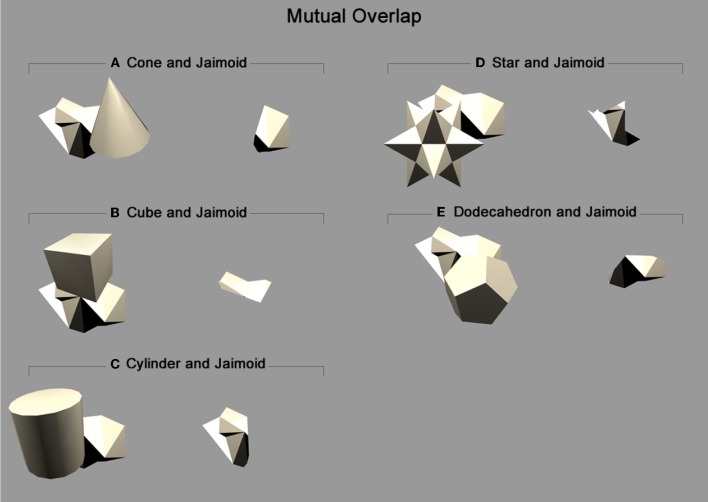
**Mutual overlap: Areas of mutual overlap between the occluding and occluded objects during one example frame of rotation.** As the two objects rotate together in lock-step, the area of mutual overlap creates a partial view of the occluded object: **(A)** Shows the cone and Jaimoid; **(B)** Cube and Jaimoid; **(C)** Cylinder and Jaimoid; **(D)** Star and Jaimoid; **(E)** Dodecahedron and Jaimoid. Each pair is presented alongside the partial view it creates. VisNet must learn to associate together all of the different partial views of the occluded object to build an exclusively invariant representation.

**Figure 11:**
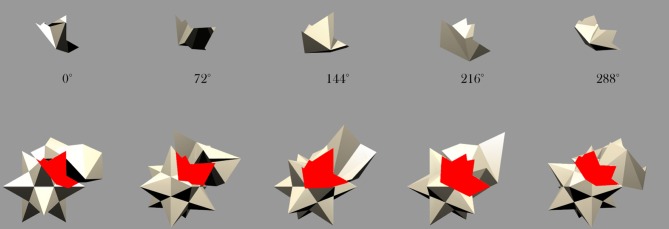
**Star and Jaimoid mutual overlap: Five example frames of the star and Jaimoid as they rotate together in lock-step.** As in Figure 10, the area of mutual overlap creates a partial view of the Jaimoid. This is shown in this specific example where the star and Jaimoid are presented over five equally spaced viewing angles.

Finally, VisNet was also tested with the different partial views of the occluded object as presented in Figure 10 and exemplified in Figure 11. As the different object pairs rotate together over 360°, parts of the partially occluded object are not visible. By testing VisNet with the partial views that were not visible during training it is possible to establish whether VisNet is able to bind together the partially occluded views of the occluded object into one holistic invariant representation. This test is important because it shows that VisNet does not need to rely on a key component of the training stimuli in order to recognise it.

The network's ability to recognise which object is shown during testing is assessed using two information theoretic measures: single and multiple cell information. Full details on the application of these measures to VisNet are given by Stringer et al. (2006). These measures reflect the extent to which cells respond invariantly to an object over a number of different views (transforms), but respond differently to different objects. The single cell information measure is applied to individual cells in layer 4 of the VisNet model, and measures how much information is available from the response of a single cell about the stimlus that was presented. The single cell information measure for each cell shows the maximum amount of information that the cell conveys about any one object. This is computed using the following formula with details provided by Rolls et al. (1997) and Rolls and Milward (2000). The object-specific information *I*(*s*, *R*) is the amount of information the set of responses *R* has about a specific object *s*, and is given by

(7) $$I(s,R)=\sum_{r\in R}P(r|s)\log_{2}\frac{P(r|s)}{P(r)},$$

where *r* is an individual response from the set of responses *R*. However, the single cell information measure cannot give a complete assessment of VisNet's performance with respect to invariant object recognition. If the amount of information provided by a single cell is not sufficient to differentiate between which objects are present during testing, the network may have failed to learn, or a distributed representation may have formed that needs information from a population of neurons to encode which object is present. Furthermore, if all output cells learned to respond to the same object then there would in fact be relatively little information available about the set of objects *S*, and single cell information measures alone would not reveal this. To address these issues, we also calculate a multiple cell information measure, which assesses the amount of information that is available about the whole set of objects from a population of neurons.

Procedures for calculating the multiple cell information measure are described in detail by Rolls et al. (1997) and Rolls and Milward (2000). From a single presentation of an object, we calculate the average amount of information obtained from the responses of all the cells regarding which object is shown. This is achieved through a decoding procedure that estimates which object *s*′ gives rise to the particular firing rate response vector on each trial. A probability table of the real objects s and the decoded objects *s*′ is then constructed. From this probability table, the mutual information is calculated as

(8) $$I(S,S^{\prime })=\sum_{s,s^{\prime }}P(s,s^{\prime })\log_{2}\frac{P(s,s^{\prime })}{P(s)P(s^{\prime })}.$$

Multiple cell information values are calculated for the subset of cells which, according to the single cell analysis, have the most information about which object is shown. In particular, the multiple cell information is calculated from the first five cells for each object that had the most single cell information about that object. This results in a population of 30 cells given that there were six objects. Previous research (Stringer and Rolls, 2000) found this to be a sufficiently large subset to demonstrate that invariant representations of each object presented during testing were formed, and that each object could be uniquely identified.

## 3. Results

### 3.1. Analysis of individually rotating objects

After the network had been trained on pairings of the occluded and five occluding objects, we tested whether the network had built transform invariant representations of the objects through a CT learning effect. By presenting the rotating objects individually (Figure 4) to the network we were able to record the cell response properties of the neurons in the fourth layer of VisNet for each of the objects. A large number of individual experiments were performed across different parameters and random seeds to ensure the consistency and validity of the results. However, the results presented are all collected as part of the same individual experiment.

**Figure 12:**
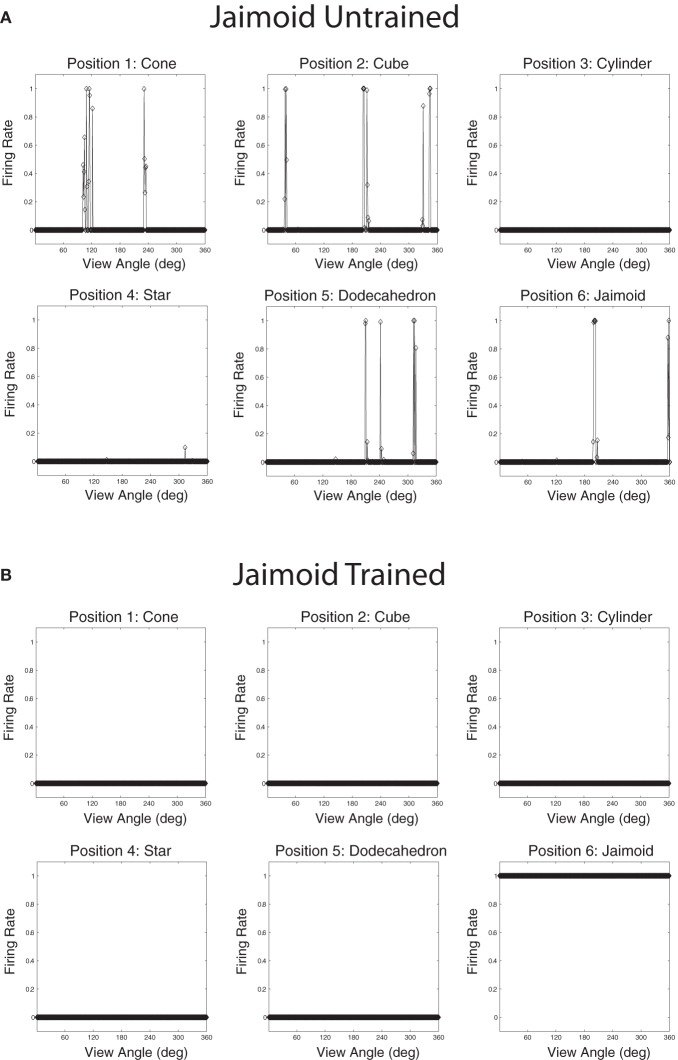
**The firing rate responses of cell (4, 17) in the 4th (output) layer of VisNet to the central occluded object (Jaimoid) and the five surrounding occluding objects as they rotated through 360° in 1° steps before and after training.** Before training, it can be seen that the cell responds randomly to different views of different objects. After training, it can be seen that the cell's response pattern has changed. This cell responds to all the views of Jaimoid, and to none of the views of the other objects.

Populations of cells that responded invariantly to the individual objects were found. These cells responded to only one object and to no views of any of the other objects. Figures 12A,B show the cell response plots for cell (4, 17), selected at random, as each object is rotated through 360° in 1° steps. Figure 12A shows the responses of the cell before training and Figure 12B shows the cell responses after training. The six response plots of cell (4, 17) before training show that the cell responds at random to the six objects. After training, the cell has learned to respond to the central occluded object, the Jaimoid, invariantly and does not respond to any view of any of the other objects.

**Figure 13:**
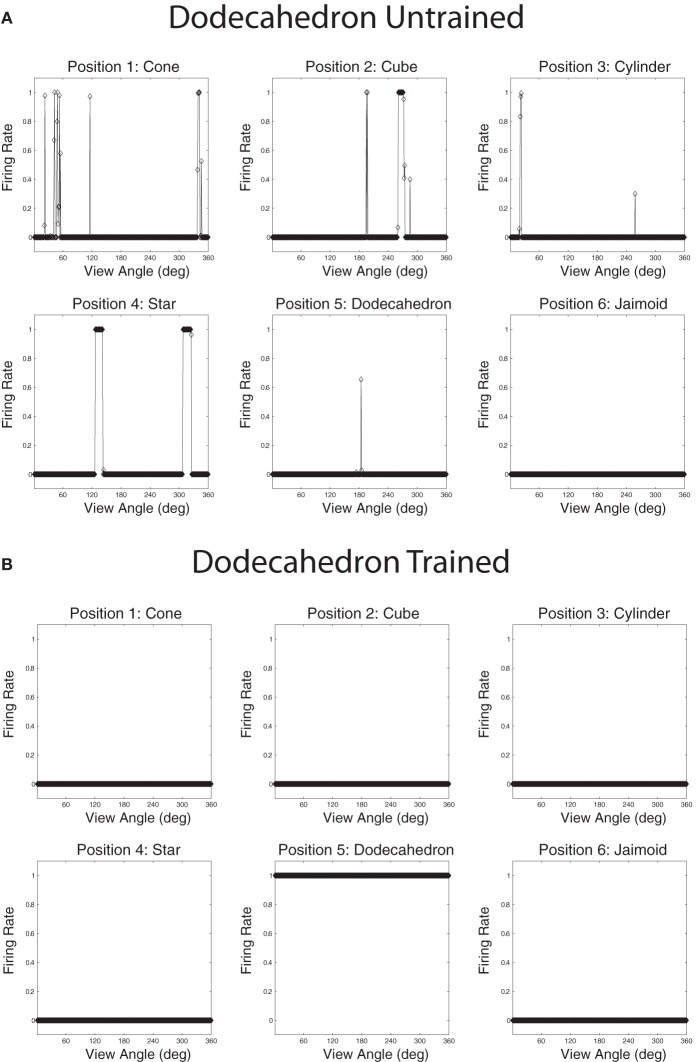
**The firing rate responses of cell (19, 1) in the fourth (output) layer of VisNet to the central occluded object (Jaimoid) and the five surrounding occluding objects as they rotated through 360° in 1° steps before and after training.** Before training it can be seen that the cell responds randomly to different views of different objects. After training it can be seen that the cell's response pattern has changed. This cell has become an exclusive invariant cell for the Dodecahedron. It responds invariantly to all 360 views of the Dodecahedron and to no views of any other object.

Figures 13A,B show the cell response plots for cell (19, 1) before and after training, respectively. Before training, the cell responds to the objects randomly. After training this cell has learned to respond invariantly to all 360 views of the Dodecahedron, which was one of the occluding objects, and to no views of any other objects. Furthermore, although not shown here, other output cells learned to respond in a selective and invariant manner to each of the other occluding objects. Thus, all of the objects were represented individually. When different sparseness values throughout the layers were investigated, results were found to be robust. As the sparseness was gradually increased, a more distributed representation began to form with fewer exclusive cells whereby each cell began to respond to more than one object. In this situation, object identity is still encoded but over a population of cells.

### 3.2. Analysis of cell firing properties in earlier layers

The analyses described above were applied to the output (fourth) layer of the network. Cells in the output layer receive information through the feedforward synaptic connections from across the entire input retina. However, cells in the earlier layers receive more localized input from the retina due to the topographical feedforward connectivity present within the model. Therefore, we carried out additional analyses of the cell response properties in the earlier layers after training.

**Figure 14:**
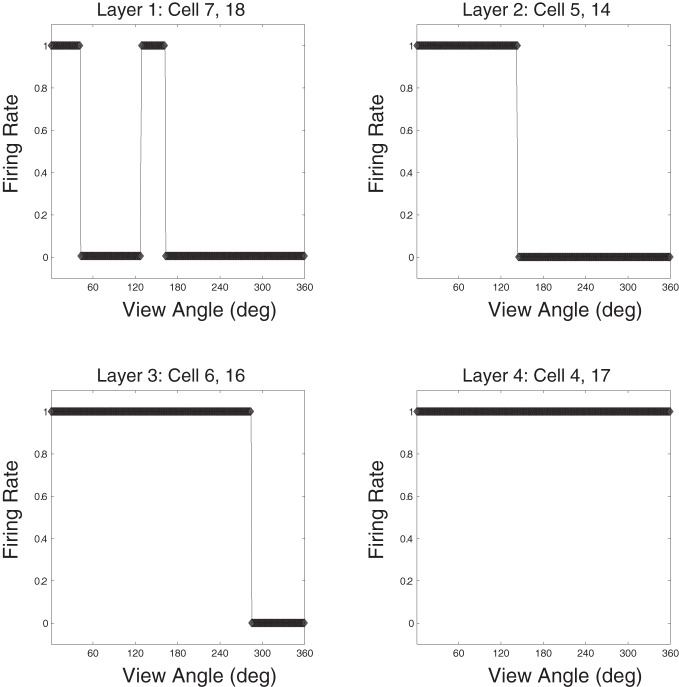
**Example Jaimoid response plots for all four layers.** A prototypical example cell is presented for each of the 4 layers within the VisNet model. For each cell, its response plot is presented with respect to the example object, the Jaimoid. It can be seen that a typical layer 3 cell is highly transform invariant while a layer 2 cell shows more modest levels of view invariance. Layer 1 cells demonstrate very little view invariance and responded to a very narrow set of views. In all cases, these cells responded exclusively to their preferred object, in this case the Jaimoid, and did not learn to respond to any views of any of the other objects. Comparable responses exist for all six of the objects presented during training.

Response plots for cells that have learned to respond to the Jaimoid are presented for each of the four layers in Figure 14. It was found that the responses of cells in layer 3 of the network were similar to those in the output (fourth) layer. That is, cells in layer 3 were both object-selective (responding exclusively to their preferred object) and highly transform (view) invariant. In layer 2 of the network, cells were object-selective but showed more modest levels of transform invariance due to the limited convergence of feedforward connections from the retina. Individual layer 1 cells receive projections from a very limited region of the retina. These cells were object-selective but provided very low levels of transform invariance. Stringer and Rolls (2008) have shown that a one-layer network with full feedforward connectivity can learn output representations that are object-selective and completely transform invariant, even when trained on pairs of objects simultaneously. Although, these authors did not look at the case of realistic visual objects that are partially occluding during training.

### 3.3. Analysis of partial view response

To better understand how the network has learned to represent the partially occluded object, the different fragmented partial views of the occluded object that were obscured at different times during training were presented separately to the model during testing for all 360 views. This is a fundamental test to ensure that output neurones have learned to respond to the fragmented parts of the partially occluded object. A similar but easier test would have been to only present the two different halves of the Jaimoid to the network for testing. By presented the smaller fragmented views, it may shown that VisNet has successfully learned to bind these partial views together form a complete invariant representation of the Jaimoid. This test is necessary to show that output neurons do not just relying on a key-marker or partial view of the Jaimoid in order to recognise it.

**Figure 15:**
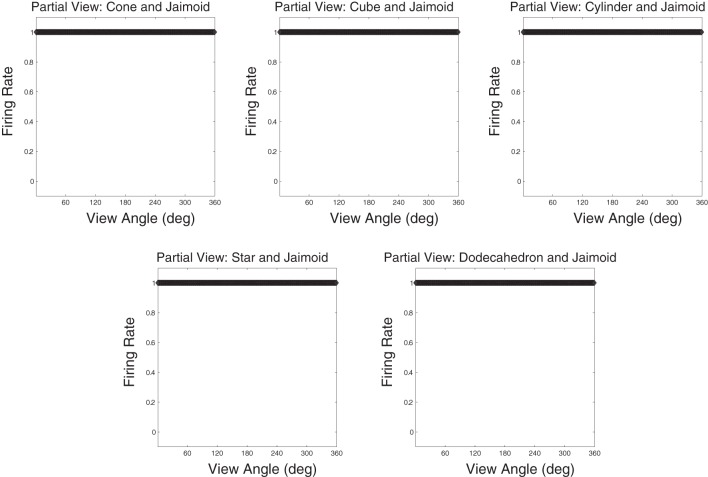
**Partial view response plots for cell 4, 17.** Cell response plots are presented after testing the network with the fragmented partial views of the Jaimoid, as exemplified in Figure 10. It can be seen that the example cell 4, 17 that responded invariantly to the complete view of the Jaimoid also responds in an identical manner to the different fragmented partial views of the Jaimoid. Cell 4, 17 has learned to bind together these different partial views into a holistic representation and responds equally well to all of them, thus proving that this cell does not rely on a specific partial view or key-marker in order to recognize the Jaimoid.

Figure 15 shows that neurons in the output layer of the VisNet model were able to successfully bind together all of the different partial views into a holistic invariant representation. Specifically, the exact same output neurons (e.g., cell 4, 17) that responded invariantly when presented with the complete Jaimoid (e.g., Figure 12) were also activated in an identical manner when presented with the various partial views. Each and every partial view caused the same output neurons to respond invariantly as if the whole object had been presented in its entirety.

This important novel result shows that a feedforward hierarchical model of the ventral visual system such as VisNet does not need to rely on particular parts, or key-markers, of an object in order to recognize it. Furthermore, it shows that such a biologically inspired network is able to not only build invariant representation of individual objects despite the fact that pairs of objects were presented during training, but it also shows that such a network can solve a far more complex problem, that is, building invariant representations in a multi-object environment even when the different objects are partially occluding one another, as is often the case in the real world.

### 3.4. Location versus object selectivity

An important question is whether the output cells learned to respond to the visual form of the objects or merely to the retinal locations. In order to minimize the possibility that the output cells had learned to respond to the locations, the objects were presented to the network in overlapping locations as shown in Figure 8. Even with the objects presented in highly overlapping locations, the output cells learned to respond to the objects themselves, and to no views of any of the other partially overlapping objects.

**Figure 16:**
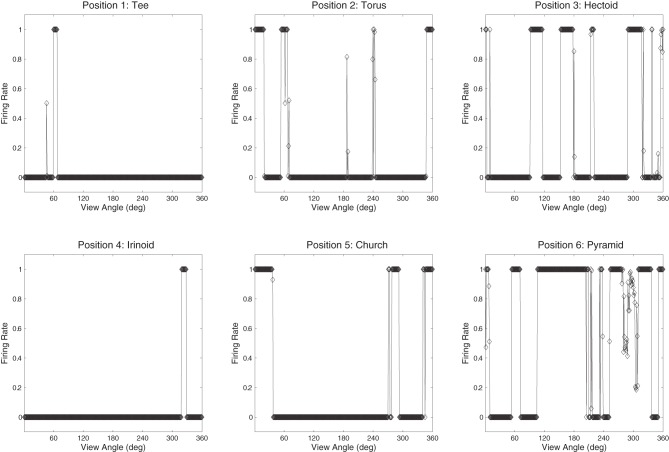
**The firing rate responses of cell (4, 17) in the fourth (output) layer of VisNet to six new objects that the network was not trained on, as they rotated through 360° in 1° steps after training.** It can be seen that the cell's response pattern has changed compared to its response pattern to the six objects that the network was trained on (the occluded and the five occluding objects; Figure 12B). Whereas when tested with the set of objects that the network was trained on, the cell responds to at least 80% of the views of Jaimoid, and to none of the views of the other objects. When the network is tested on six novel objects presented in the same locations as the trained set of objects, the cell responds very poorly to small portions of view of a number of objects. This shows that the network has learnt to respond to the trained objects in particular, and not just to their locations.

**Figure 17:**
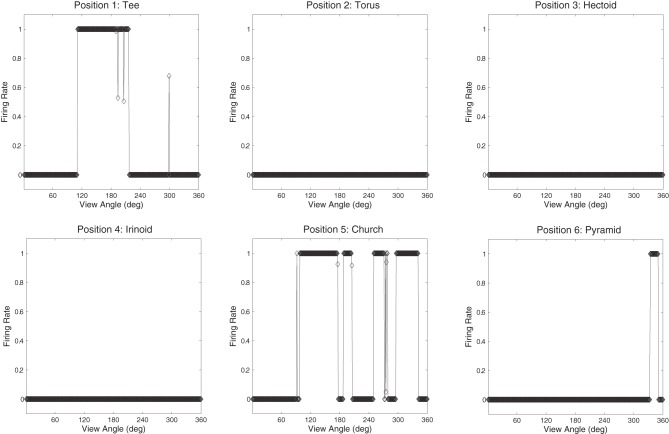
**The firing rate responses of cell (19, 1) in the fourth (output) layer of VisNet to six new objects that the network was not trained on, as they rotated through 360° in 1° steps after training.** It can be seen that the cell's response pattern has changed compared to its response pattern to the six objects that the network was trained on (the occluded and the five occluding objects; Figure 13B). Whereas when tested with the set of objects that the network was trained on, the cell responds invariantly to all 360 views of the Dodecahedron and to no views of any other object. When tested on the six novel objects presented in the same locations as the trained set of objects, the cell responds very poorly to small portions of view of a number of objects. This shows that the network has learnt to respond to the trained objects in particular and not just to their locations.

The network was also tested with six novel objects, such as a pyramid, rotating over 360° in 1° steps. These objects are novel in the sense that the network was not trained with them and was only exposed to them for testing. The novel objects were presented in the same locations as the original occluding and occluded objects. If the network had learnt to respond to the individual trained objects rather than the locations, then the responses of the output cells to the novel objects should be less clearly tuned than to the trained objects. That is, the cells should not respond so uniformly (invariantly) over the different views of any particular novel object, and the cell responses should not be selective to individual novel objects. Figures 16 and 17 show cell response plots for cell (4, 17) and (19, 1) after testing the network with the novel objects. To reiterate, when tested with the original set of objects, cell (4, 17) responds invariantly to the Jaimoid, and to none of the views of the other objects (Figure 12B). However, when the network is tested on six novel objects presented in the same locations as the trained set of objects, the cell responds very poorly to small portions of view of a number of objects. Similarly, when tested with the original set of objects, cell (19, 1) responds invariantly to all 360 views of the Dodecahedron and to no views of any other object (Figure 13B). When tested on the six novel objects presented in the same locations as the trained set of objects, the cell responds very poorly to small portions of view of a number of objects. These results help demonstrate that the network has learnt to respond to the trained objects in particular, and not just to their locations.

### 3.5. Information analysis

**Figure 18:**
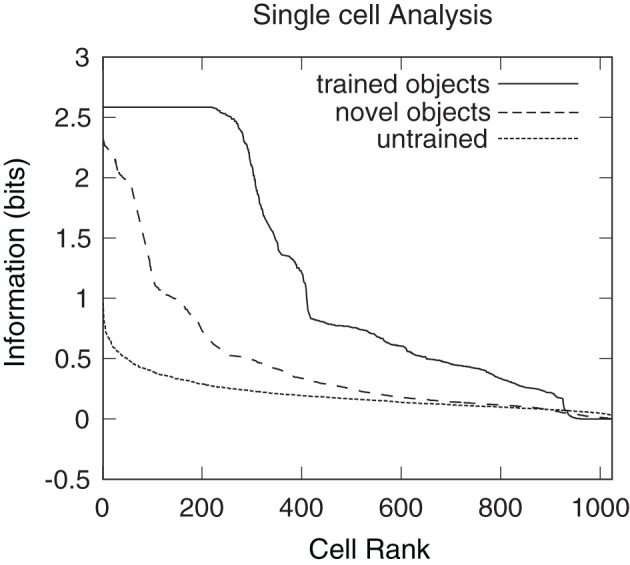
**Single cell information results obtained when VisNet was tested with the occluded object and five occluding objects rotating through 360° in 1° steps.** Single cell information analysis results are also plotted for six novel objects that the network was not previously trained on. These novel objects were rotating in the same positions as the six objects with which the network was originally trained. Results are presented having tested the trained network with the original set of trained objects (unbroken line), after testing the trained network with the novel set of objects (dashed line), and after testing a random untrained network with the original six objects (dotted line). The single cell information measure for all fourth layer neurons ranked in order of their invariance to the objects is shown. It can be seen that training the network on the object pairs has led to many fourth layer neurons attaining the maximum level of single cell information of 2.58 bits for these trained objects. These cells have learned to respond selectively to individual trained objects invariantly over all views. It can also be seen that the novel objects produce less information and no cells reached the maximal information. The random untrained plot provides a baseline comparison.

Single cell information analysis was conducted to confirm whether the network had developed cells that responded invariantly to their preferred object (Figure 18). The unbroken line represents the results obtained after presenting the six original trained objects to a network after training on the 360 views of all possible object pairs. The dashed line represents the results obtained after presenting six novel objects rotating in the same positions as the six original trained objects. The dotted line represents the results after presenting the six original objects to a random untrained network. Single cell information measures for the fourth layer neurons ranked in order of their invariance to the objects are shown. It can be seen that training the network on the object pairs has lead to many of the fourth layer neurons attaining the maximal level of single cell information of 2.58 bits for the trained objects. These neurons have learned to respond to all of the views of their preferred object. However, when the network, which had been trained on the six orginal objects, was tested with novel objects, no cells reached the maximum level of information. This reflected the fact that the output cells of the trained network were not able to respond to the novel objects in a view-invariant or object-selective manner, as shown in Figures 16 and 17. These results thus further demonstrate that when the network was trained on the six orginal objects, the output cells had learned to respond selectively to the trained objects and not the untrained objects. This in turn confirms that the network learned to respond to the visual forms of the trained objects rather than their retinal locations.

However, it is unclear whether all of the six objects are individually represented by a unique subset of invariant output cells. Indeed, it is possible that these cells are responding to the same object and are, therefore, unable to provide information regarding which object is present. To ensure that there are cells that respond preferentially to each of the six objects multiple cell information analysis was performed.

**Figure 19:**
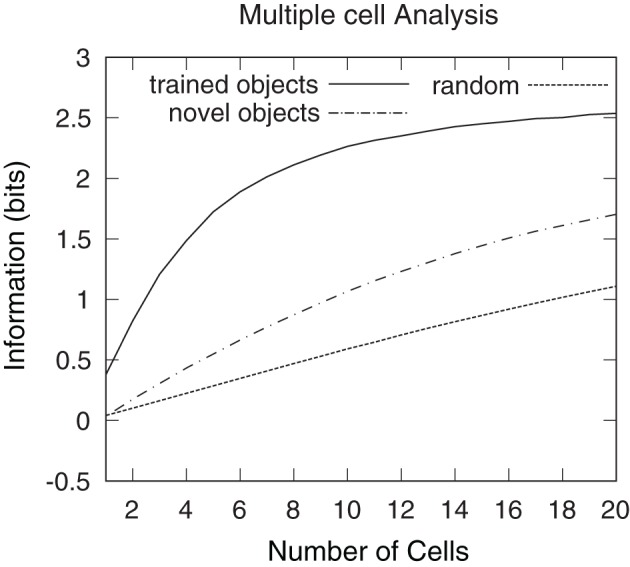
**Multiple cell information results obtained when VisNet was tested with the occluded object and five occluding objects rotating through 360° in 1° steps.** Multiple cell information analysis results are also plotted for six novel objects that the network was not previously trained on. These novel objects were rotating in the same positions as the six objects on which the network was originally trained. Results are presented after training the network (unbroken line), after testing the trained network with the novel set of objects (dashed line) and with a random untrained network (dotted line). After the network was trained, over 2.5 bits of information was reached, which was substantially higher than 1.10 bits reached by the untrained network or 1.7 bits reached by the trained network that was tested on the novel set of objects. This confirmed that, after training the network, there were cells that form object specific representations to each one of the six objects and do not respond to the object locations.

Figure 19 shows the multiple cell information analysis obtained when VisNet was tested with the six individual objects rotating through 360° in 1° steps. Multiple cell information analysis results are also plotted for six novel objects that the network was not previously trained on. These novel objects were rotating in the same positions as the six objects on which the network was originally trained. Results are presented having tested the trained network with the original set of objects (unbroken line), after testing the trained network with the novel set of objects (dashed line) and with a random untrained network (dotted line). After the network was trained and tested with the original set of objects, over 2.5 bits of information was reached (substantially higher than 1.1 bits reached by the untrained network or 1.7 bits reached by the trained network that was tested on the novel set of objects) suggesting that the single cell information results included cells that preferentially responded to all six objects. These plots show that the network did not learn to respond to the locations of the objects, and instead bound together different views of the occluding and occluded object to form object specific representations. This was also confirmed by inspection of the cell response plots as shown in Figures 12B and 16, as well as Figures 13B and 17.

## 4. Discussion

An important question in natural vision is how the brain forms invariant representations of objects that are always partially occluded by other objects during learning. In a real world visual environment, this will often be the case. Stringer and Rolls (2008) have shown that a biologically plausible competitive neural network (VisNet) can develop invariant representations of individual objects when no single object is seen in isolation. In this paper we demonstrate for the first time how such a network might form an invariant representation of an object that is always partially occluded by other objects. The mechanism employed for invariance learning is CT learning. CT learning uses the spatial continuity between the views of individual objects as they transform in the real world, combined with associative learning of feedforward connection weights.

It was found that, after training the network with a rotating object that is always partially occluded, the network is able to form view invariant representation of the partially occluded object. In addition, by testing the network with the fragmented partial views of the occluded object in isolation, it was also shown that the same output neurons that learned to respond to the Jaimoid when presented in its entirety also responded in an identical manner to each of the fragmented partial view sequences. This shows that the network has learned to bind together the different partial views of the occluded object presented during training into a holistic invariant representation despite always seeing the Jaimoid partially occluded and, therefore, never in isolation. It was also found that view invariant representations are also formed for all five occluding objects. This is a challenging task since the occluding objects were always overlapping the occluded object and therefore VisNet had to learn to separate the objects. This is the first time such learning has been shown to happen in a biologically inspired model of the ventral visual system.

Despite the fact that the objects were presented in the same location during training and testing, the network was able to form representations of the objects' identities instead of just learning to respond to particular locations. During training there was significant overlap between the objects (Figures 8 and 9), which would have precluded VisNet from learning about each object just because it was presented in the same location. Instead, VisNet built separate representations of each of the individual objects. This is confirmed by invariant cells that responded maximially to all views of only one of the stimuli and not to any views of any other stimuli. After training, all of the stimuli were represented in this way. Given that the objects were highly overlapping during training, if the network had learned to respond to location instead of stimulus identity, then the network would not have developed cells which responded specifically and invariantly to individual objects, with all of the objects represented uniquely in this way.

This conclusion is also confirmed by additional results obtained with a novel set of six objects presented during testing. These novel objects were presented in the exact same locations as the six original objects that were presented during training. The output neurons that learned to respond preferentially and invariantly to the original trained objects were then inspected and an example response plot was presented. It was shown that these output neurons did not respond in a exclusive or invariant manner to any of these novel objects thus confirming that the network had learned object selectivity. The residual firing that was present within the response plots can be explained by the fact that after the network has been trained, neurons will respond in proportion to how similar the novel input pattern is to the previous learned patterns. By virtue of the fact that the input spaces are not orthogonal with respect to the input filters that they activate when objects occupy the same input space on the retina, they will cause an degree of activity in the network based on previous learning. This is a fundamental property of competitive networks, which will try to generalize to novel input patterns depending on their similarity to the previous learned input patterns.

Most artificial computer vision systems designed by software engineers do not seek to mimic processing exactly as it is carried out in the brain. Also, the challenges addressed by artificial computer vision systems are often more focused, for example, on the problem of object or face recognition after training the system with individual segmented objects or faces. For such a task, non-biologically inspired artificial visual systems often rely on either template matching or searching for the presence of a subset of key features in order to recognise a partially occluded object (Ullmann, 1992; Ying and Castañon, 2002; Do et al., 2005). However, as computational neuroscientists, we are interested in the more ecological problem of understanding how the primate visual system learns in an unsupervised manner to make sense of complex natural visual scenes containing multiple objects. This is a valuable long term goal, which may ultimately offer engineers powerful new approaches to intelligent visual scene analysis and object recognition. Understanding how the primate brain learns to process visual input from scenes will involve the step-by-step uncovering of many key neurodynamical mechanisms, which ultimately blend and work together in the brain. In this current paper, we have examined the problem of how the primate visual system might develop separate transform-invariant representations of individual objects even if these objects are always seen partially occluding each other during unsupervised learning. The simulations reported here have shown for the first time how a biologically plausible model, VisNet, of the ventral visual pathway, with a Hebbian associative synaptic learning rule, is able to solve this particular problem.

### 4.1. Future work

The results described above have shown how a biologically plausible neural network model of the ventral visual pathway, VisNet, is able to develop object-selective and rotation-invariant representations of objects that were partially occluding each other during training. However, more work needs to be done to explore the limits of this mechanism.

For example, what proportion of an object needs to be visible? Future research could investigate the exact degree to which objects can be occluded before learning of the partially occluded object breaks down. This avenue of research could address the effect of occlusion by two or more objects at a time, or where there is more than one occluded object. The results of these experiments presented within this study suggest that the extent to which the partially occluded object is covered could increase quite considerably so long as the different parts of the occluded object have all become visible at some point during training.

The use of simple geometric shapes is a limitation of the current study that should be addressed as part of future research. The choice to use simple geometric shapes was not to help VisNet solve the task at hand. These shapes allowed for a level of control necessary to answer the question “how” has VisNet solved this problem. This level of control is harder to achieve with more complex objects, but their use is a sensible next step to explore their effect on the self-organization of the network. The authors believe that the types of objects used will not have any qualitative impact on the results presented within this paper, but this should be confirmed. So long as the resolution of the retina is high enough to convey the necessary detail of the more natural objects, then the VisNet model will make use of the same principles discussed within this study to solve the problem.

In the simulations described above, individual objects rotated on the same part of the retina. Perhaps a more challenging problem is the *translation* of objects across the retina. This would happen naturally as an observer shifts their gaze around a visual scene. In this case, all of the objects would be seen moving over the entire retina. The input representations of the objects would then fully overlap over all possible locations on the retina. Yet the network must still form separate output representations of the objects, which are also translation invariant. We hypothesise that the network described in this paper should still be able to solve this problem using similar learning principles.

Another limitation of the current study is that it explores only one type of invariance learning mechanism, CT learning (Stringer et al., 2006). This binds different transforms of a particular object together by exploiting the spatial similarity that exists between the different transforms of that object. As discussed, CT learning relies on a simple Hebbian learning rule. It would be very interesting to investigate if similar results can be achieved with a different type of biologically plausible learning rule such as Trace learning (Foldiak, 1991; Wallis and Rolls, 1997). This alternative learning rule exploits temporal continuity of successive transforms of an object in order to build a transform-invariant representation of that object. Trace learning utilizes a memory trace of the recent firing of the post-synaptic cell.

In natural vision, objects are not always moving with respect to one another, nor with respect to the viewer. Sometimes objects are simply static, and one object will occlude another and yet in many situations we are still able to learn to recognize the partially occluded object. A typical situation of this kind might occur when we view some faces in a photograph, for example. The current VisNet model would not be able to solve such a training paradigm because it relies on the statistical decoupling of features between different objects that can occur through independent movement in order to tell them apart. However, our laboratory has recently shown that this more difficult problem can be solved using spiking neural network dynamics. In such a model, the times of individual action potentials are simulated, and the synaptic plasticity can be dependent on the times of the pre- and post-synaptic spikes (Bi and Poo, 1998).

### Conflict of interest statement

The authors declare that the research was conducted in the absence of any commercial or financial relationships that could be construed as a potential conflict of interest.

## References

[B1] Bi G. G. Poo M. M. (1998). Synaptic modifications in cultured hippocampal neurons: dependence on spike timing, synaptic strength, and postsynaptic cell type. J. Neurosci. 18, 10464–10472 9852584 10.1523/JNEUROSCI.18-24-10464.1998 PMC6793365

[B2] Booth M. C. Rolls E. T. (1998). View-invariant representations of familiar objects by neurons in the inferior temporal visual cortex. Cereb. Cortex 8, 510–523 10.1093/cercor/8.6.510 9758214

[B3] Desimone R. (1991). Face-selective cells in the temporal cortex of monkeys. J. Cogn. Neurosci. 3, 1–8 10.1162/jocn.1991.3.1.1 23964801

[B4] Do Q. V. Lozo P. Jain L. C. (2005). A Vision System for Partially Occluded Landmark Recognition, Vol. 3809/2005 Berlin/Heidelberg: Springer

[B5] Foldiak P. (1991). Learning invariance from transformation sequences. Neural Comput. 3, 194–200 10.1162/neco.1991.3.2.194 31167302

[B6] Hasselmo M. E. Rolls E. T. Baylis G. C. Nalwa V. (1989). Object-centered encoding by face-selective neurons in the cortex in the superior temporal sulcus of the monkey. Exp. Brain Res. 75, 417–429 2721619 10.1007/BF00247948

[B7] Hawken M. J. Parker A. J. (1987). Spatial properties of neurons in the monkey straite cortex. Proc. R. Soc. Lond. B. Biol. Sci. 231, 251–288 2889214 10.1098/rspb.1987.0044

[B8] Hertz J. Krogh A. Palmer R. G. (1991). Introduction to the Theory of Neural Computation. Workingham, UK: Addison Wesley

[B9] Ito M. Tamura H. Fujita I. Tanaka K. (1998). Size and position invariance of neu-ronal response in monkey inferotemporal cortex. J. Neurophysiol. 73, 218–226 7714567 10.1152/jn.1995.73.1.218

[B10] Kobatake E. Tanaka K. (1994). Neuronal selectivities to complex object features in the ventral visual pathway of the macaque cerebral cortex. J. Neurophysiol. 71, 856–867 8201425 10.1152/jn.1994.71.3.856

[B11] Op De Beeck H. Vogels R. (2000). Spatial sensitivity of macaque inferior temporal neurons. J. Comp. Neurol. 426, 505–518 10.1002/1096-9861(20001030)426:4<505::AID-CNE1>3.0.CO;2-M 11027395

[B12] Perrett D. I. Oram M. W. (1993). Neurophysiology of shape processing. Image Vis. Comput. 11, 317–333

[B13] Perry G. Rolls E. T. Stringer S. M. (2006). Spatial vs temporal continuity in view invariant visual object recognition learning. Vision Res. 46, 3994–4006 10.1016/j.visres.2006.07.025 16996556

[B14] Rolls E. T. (1992). Neurophysiological mechanisms underlying face processing within and beyond the temporal cortical visual areas. Philos. Trans. R. Soc. Lond. B Biol. Sci. 335, 11–20 10.1098/rstb.1992.0002 1348130

[B15] Rolls E. T. (2000). Functions of the primate temporal lobe cortical visual areas in invariant visual object and face recognition. Neuron 27, 205–218 10.1016/S0896-6273(00)00030-1 10985342

[B16] Rolls E. T. Deco G. (2002). Computational Neuroscience of Vision. Oxford, UK: Oxford University Press

[B17] Rolls E. T. Milward T. (2000). A model of invariant object recognition in the visual system: learning rules, activation functions, lateral inhibition, and information-based performance measures. Neural Comput. 12, 2547–2572 11110127 10.1162/089976600300014845

[B18] Rolls E. T. Stringer S. M. (2001). Invariant object recognition in the visual system with error correction and temporal difference learning. Network 12, 111–129 11405418

[B19] Rolls E. Treves A. (1990). The relative advantages of sparse versus distributed encoding for associative neuronal networks in the brain. Network 1, 407–421

[B20] Rolls E. T. Treves A. (1998). Neural Networks and Brain Function, 1st Edn Oxford: Oxford University Press

[B21] Rolls E. T. Treves A. Tovee M. J. Panzeri S. (1997). Information in the neuronal representation of individual stimuli in the primate temporal visual cortex. J. Comput. Neurosci. 4, 309–333 10.1023/A:1008899916425 9427118

[B22] Royer S. Pare D. (2003). Conservation of total synaptic weight through balanced synaptic depression and potentiation. Nature 422, 518–522 10.1038/nature01530 12673250

[B23] Stringer S. M. Rolls E. T. (2000). Position invariant recognition in the visual system with cluttered environments. Neural Netw. 13, 305–315 10.1016/S0893-6080(00)00017-4 10937964

[B24] Stringer S. M. Perry G. Rolls E. T. Proske J. H. (2006). Learning invariant object recognition in the visual system with continuous transformations. Biol. Cybern. 94, 128–142 10.1007/s00422-005-0030-z 16369795

[B25] Stringer S. M. Rolls E. T. (2008). Learning transform invariant object recognition in the visual system with multiple stimuli present during training. Neural Netw. 21, 888–903 10.1016/j.neunet.2007.11.004 18440774

[B26] Stringer S. M. Rolls E. T. Tromans J. M. (2007). Invariant object recognition with trace learning and multiple stimuli present during training. Network 18, 161–187 10.1080/09548980701556055 17966074

[B27] Tanaka K. Saito H. Fukada Y. Moriya M. (1991). Coding visual images of objects in the inferotemporal cortex of the macaque monkey. J. Neurophysiol. 66, 170–189 1919665 10.1152/jn.1991.66.1.170

[B28] Tovee M. J. Rolls E. T. Azzopardi P. (1994). Translation invariance in the responses to faces of single neurons in the temporal visual cortical areas of the alert macaque. J. Neurophysiol. 72, 1049–1060 7807195 10.1152/jn.1994.72.3.1049

[B29] Ullmann J. R. (1992). Analysis of 2-D occlusion by subtracting out. IEEE Trans. Pattern Anal. Mach. Intell. 14, 485–489

[B30] Wallis G. Rolls E. T. (1997). Invariant face and object recognition in the visual system. Prog. Neurobiol. 51, 167–194 10.1016/S0301-0082(96)00054-8 9247963

[B31] Wallis G. Rolls E. T. Foldiak P. (1993). Learning invariant responses to the natural transformations of objects. Int. Jt. Conf. Neural Netw. 2, 1087–1090

[B32] Ying Z. Castañon D. (2002). Partially occluded object recognition using statistical models. Int. J. Comput. Vis. 49, 57–78

